# Quantitative assessment of LV function and volumes with 3-slice segmentation of cine SSFP short axis images: our experience

**DOI:** 10.1186/1532-429X-16-S1-P384

**Published:** 2014-01-16

**Authors:** Edward Kuoy, Christopher V Nguyen, Sumudu N Dissanayake, Kari J Nelson, Pablo J Abbona, Mayil S Krishnam

**Affiliations:** 1 School of Medicine, University of California, Irvine, Orange, California, USA; 2 Cardiovascular and Thoracic Imaging, Radiological Sciences, University of California, Irvine, Orange, California, USA; 3 Radiology, University of California, Irvine, Orange, California, USA

## Background

Cardiac MRI is the gold standard for assessing cardiac function, but requires contour tracing of the left ventricular (LV) myocardium through multiple cine steady-state free precession (SSFP) short axis images. Despite advancements in semi-automated software, contour detection and manual readjustments of multiple slices throughout the cardiac cavity remains a tedious process. Our goal is to assess LV functional parameters using 3-slice segmentations in comparison to conventional multi-slice segmentation in patients with a wide range of pathologies.

## Methods

We retrospectively studied 1.5T cardiac MR images of 62 patients with various clinical indications. All patients had a stack of EKG gated segmented SSFP cine images through the LV. Semi-automated cardiac MR software (Argus) was used to trace LV contours both at multiple slices from base to apex as well as just 3 slices (base, mid and apical) by two readers. End diastolic volume (EDV), end systolic volume (ESV), stroke volume (SV), and ejection fraction (EF) were calculated using both assessment methods.

## Results

**Figure 1:**
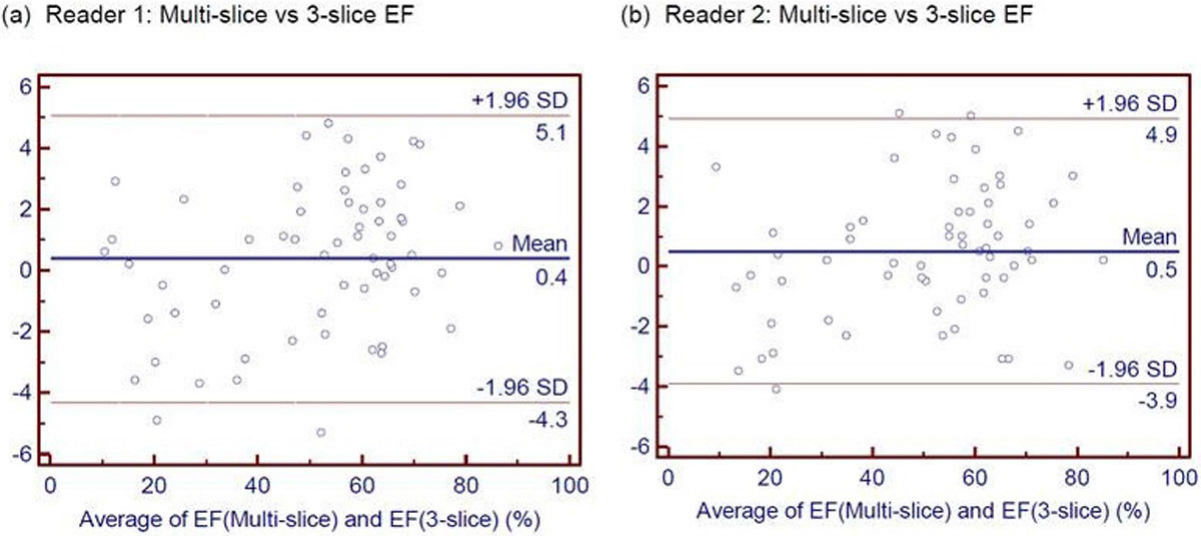
**Bland-Altman Plots for evaluation of the differences between ejection fraction (EF) measurements using multi-slice and 3-slice slice technique for (a) Reader 1 and (b) Reader 2**.

There were no statistically significant differences between EF and LV volumes obtained by multi-slice vs 3-slice analysis for both readers (P > 0.05). For reader 1 (and 2), Bland-Altman plot revealed the mean difference in LVEF between multi-slice and 3-slice analysis was 0.4% (0.5%) with limits of agreement of -4.3% to 5.1% (-3.9% to 4.9%) (Figure 1). For both readers, all differences in EF between multi-slice and 3-slice analysis were within 5.3%. The measured volumes are listed in Table 1. Multi-slice assessment required approximately 10 minutes per study while 3-slice evaluation required about 3-5 minutes.

**Table 1:** Left Ventricular Functional Parameters

	Reader 1 (3-slice)	Reader 1 (multi-slice)	Reader 2 (3-slice)	Reader 2 (multi-slice)	p-value
EF (%)	50.8 +/- 18.8	51.2 +/- 19.5	50.3 +/- 18.7	50.8 +/- 19.3	p > 0.05

EDV (mL)	112.9 +/- 55.7	117.0 +/- 59.3	113.7 +/- 54.1	118.1 +/- 57.2	p > 0.05

ESV (mL)	63.7 +/- 57.3	66.0 +/- 61.4	64.1 +/- 55.6	66.5 +/- 59.1	p > 0.05

SV (mL)	49.3 +/- 17.1	51.1 +/- 17.7	49.6 +/- 16.9	51.6 +/- 17.6	p > 0.05

CO (L/min)	3.5 +/- 1.6	3.6 +/- 1.7	3.5 +/- 1.6	3.7 +/- 1.7	p > 0.05

## Conclusions

The LVEF obtained from either 3-slice or multi-slice evaluation of SSFP cine images closely correlate across various cardiac pathologies, thereby offering a method to reduce post-processing time. Of note, although the decreased LV volumes obtained by 3-slice segmentation are statistically significant, the difference of < 3.7% may not be clinically significant.

## Funding

None.

